# A Case Report of a 3-Year-Old Child With Anaphylactic Shock After a Diclofenac Suppository Confirmed by Serial Tryptase and a Basophil Activation Test

**DOI:** 10.3389/fped.2021.802715

**Published:** 2022-02-17

**Authors:** Wun-Yan Huang, Tsu-Man Chiu, Su-Feng Kuo, Wen-Hung Chung, Yi-Giien Tsai

**Affiliations:** ^1^Department of Pediatric Emergency Medicine, Children Hospital, China Medical University, Taichung, Taiwan; ^2^Department of Medicine, School of Medicine, China Medical University, Taichung, Taiwan; ^3^Department of Dermatology, Changhua Christian Hospital, Changhua City, Taiwan; ^4^Institute of Medicine, Chung Shan Medical University, Taichung, Taiwan; ^5^Department of Laboratory Medicine, Changhua Christian Hospital, Changhua City, Taiwan; ^6^Department of Dermatology, Drug Hypersensitivity Clinical and Research Center, Chang Gung Memorial Hospital, Taipei and Linkou Branch, College of Medicine, Chang Gung University, Taipei, Taiwan; ^7^Departments of Pediatrics, Changhua Christian Children's Hospital, Changhua City, Taiwan; ^8^School of Medicine, Kaohsiung Medical University, Kaohsiung City, Taiwan; ^9^School of Medicine, Chung Shan Medical University, Taichung, Taiwan

**Keywords:** anaphylaxis, basophil activation test, non-steroidal anti-inflammatory drug, tryptase, diclofenac

## Abstract

Diclofenac is one of the most commonly used non-steroidal anti-inflammatory drug (NSAID) agents for fever management by general practitioners. Anaphylaxis due to suppository of diclofenac sodium (Voltaren) is extremely rare in children. We report the case of a 3-year-old girl with anaphylactic shock after a diclofenac suppository with confirmation by serial tryptase and a basophil activation test.

## Introduction

Anaphylaxis is an acute, potentially life-threatening allergic reaction that should be recognized promptly and managed immediately. The diagnosis of anaphylaxis is based on the history and clinical symptoms involving two or more organ systems, including the skin or mucous membranes, the respiratory system, the gastrointestinal system, and the cardiovascular system (1). The diagnosis of anaphylaxis is even more challenging in children because of the inability of children to accurately describe their symptoms and difficulties in accurately evaluating blood pressure in infants (2).

Non-steroidal anti-inflammatory drugs (NSAIDs) are considered as useful antipyretics when administered through oral or rectal routes. NSAIDs are the main culprits of drug-induced anaphylaxis, and the incidence has been reported to range from 0.04 to 3.1%, with a mortality rate of around 0.65–2% (3). Diclofenac is one of the most commonly used NSAIDs by general practitioners. The published cases of diclofenac-induced anaphylaxis include mostly oral and injectable solution, whereas rectal forms are rare (4). To our knowledge, this is the youngest child presenting with anaphylactic shock after receiving a diclofenac suppository (Voltaren). This case was confirmed by serial serum tryptase and a basophil activation test (BAT) due to its rarity and clinical importance.

## Case Report

A 3-year-old, previously healthy girl presented with sudden onset of high fever (39.1°C) at home. She had received a diclofenac suppository (Voltaren, 12.5 mg) administered by her mother without previous exposure Voltaren or similar NSAIDs. Her mother observed that she developed a pruritic erythematous rash within 30 min after receiving the diclofenac suppository. On arrival at the emergency room, she was conscious but irritable and stridor. She developed swelling of the lips and eyelids, tachycardia (>160/min), and a decrease in blood pressure (78/40 mmHg). Pulse oximetry revealed the reduction in SaO_2_ from 98 to 68%. Auscultation of her chest revealed wheeze. Two doses of 0.5 mg of epinephrine were administrated intramuscularly. This patient was also treated with crystalloid solutions, intravenous methylprednisolone, oxygen, and aerosolized salbutamol. The patient responded to this management, and the blood pressure rose to 106/52 mmHg and stridor resolved about 15 min later.

Blood sampling to laboratory testing indicated her leukocyte count to be 12,800 × 10^3^ cells/μl, and C-reactive protein was within a normal range. The patient's total IgE level was elevated at 227 IU/ml. She was only sensitivity to *Dermatophagoides pteronyssinus* IgE specific test (1.5 kU/L) using the Pharmacia CAP system (CAP) system (Pharmacia Diagnostics, Uppsala, Sweden). Acute phase value of tryptase (10.4 μg/L) was observed at the onset of anaphylaxis (ImmunoCap Tryptase, Phadia Laboratory Systems), and the value returned to the baseline range (2.7 μg/L) 24 h later. Troponins and D-dimer remained in the normal range. Non-steroidal anti-inflammatory drugs (e.g., ibuprofen) have been reported to trigger the Kounis syndrome (clinical spectrum of acute myocardial ischemia, from stable angina to acute myocardial infarction) in clinical practice (5). However, non-specific electrocardiogram abnormalities are seen in this case. Finally, human rhinovirus was detected by Real-Time RT-PCR in nasal swab.

**Figure 1:**
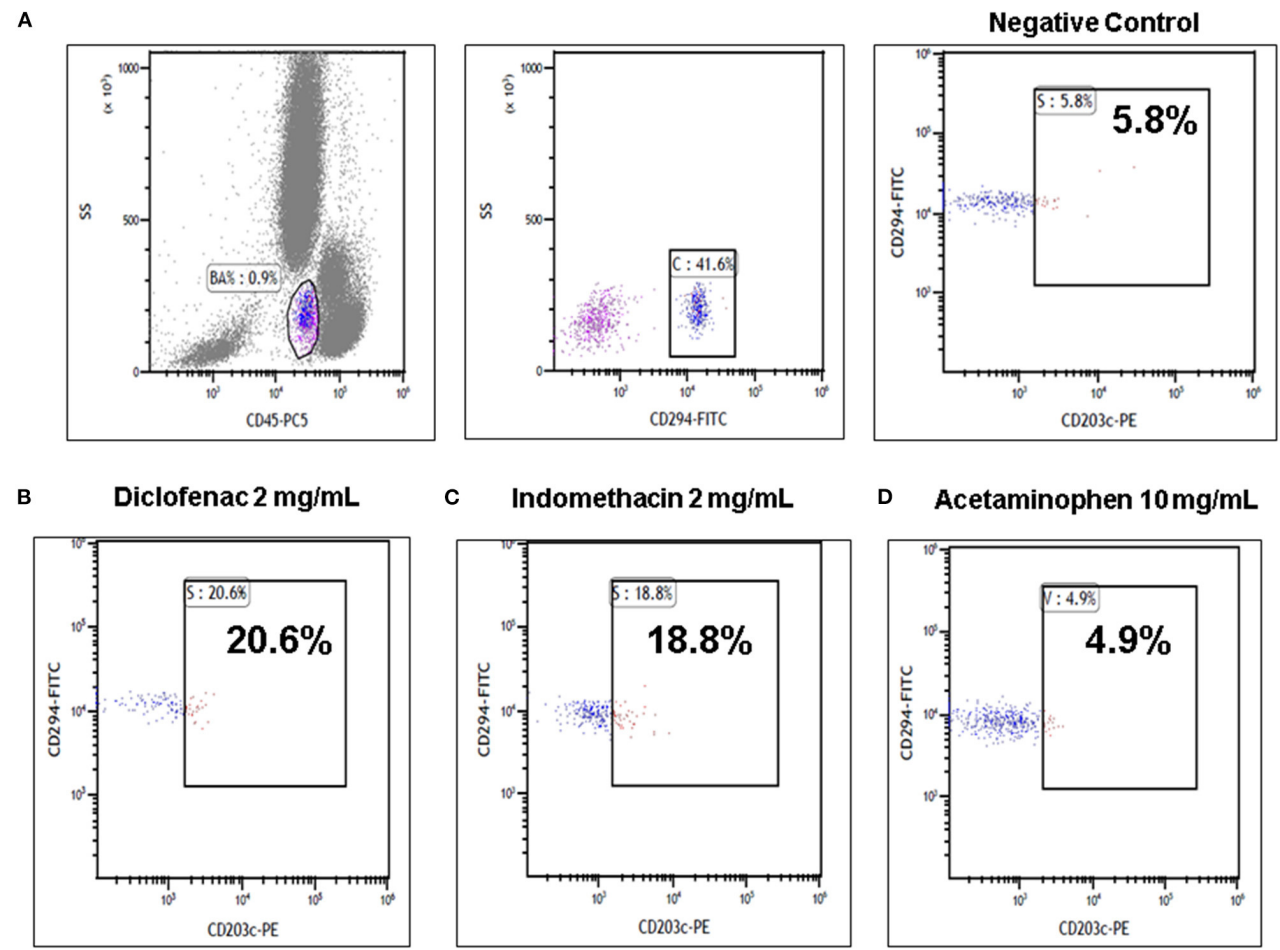
Basophil activation tests (BATs) by flow cytometry in the diclofenac anaphylactic patient. **(A)** Representative images using the basophil activation marker CD203c gating strategy showing positive results of BAT with **(B)** diclofenac (2 mg/ml) and **(C)** indomethacin (2 mg/ml) and negative results of BAT with **(D)** acetaminophen (10 mg/ml) are shown.

The parents indicated that the patient had never taken NSAIDs before and she had never experienced an adverse reaction to medications. Basophils expressing both CD45 and CD294 were examined for the CD203c expression. Leukocytes in each sample were then analyzed using a Cytomics FC 500 Flow Cytometer (Beckman Coulter Inc., Fullerton, CA, USA). If a sample shows a 5% increase in activated basophil percentage compared to the negative control, then this points to a positive result. Our study showed positive BAT results in samples with diclofenac (2 mg/ml; activated basophil: 20.6% for CD203c-PE), diclofenac (1 mg/ml; activated basophil: 12% for CD203c-PE), and indomethacin (2 mg/ml; activated basophil: 18.8% for CD203c-PE) and negative BAT results in samples with acetaminophen (10 mg/ml; activated basophil: 4.9% for CD203c-PE). At least 250 basophils were detected during basophil activated test for each test (Figure 1). Finally, the patient was diagnosed with diclofenac associated anaphylaxis and was instructed to avoid diclofenac and indomethacin according to the results of BAT. The management of anaphylaxis was smooth, and she was discharged without complication.

## Discussion

Evidences has revealed that almost 50% of diagnoses of pediatric anaphylaxis are actually missed because its symptoms imitate urticaria, and its underlying causes have been poorly investigated (6). Serum tryptase concentrations must be collected within the first 60–90 min and 24 h after the resolution of anaphylaxis to differentiate underlying mast cell disorders (7). Serum tryptase level is positively correlated with anaphylaxis severity, and it can be a useful biomarker to support the diagnosis of anaphylaxis, especially in cases of clinically ambiguous presentations (6, 7). A serum tryptase level of more than 11.4 μg/L usually suggests anaphylaxis in adult patients, but only 19.2% of severely anaphylactic children have elevated levels of tryptase above 11.4 μg/L (7, 8). The anaphylaxis working conference in 2010 proposed a consensus equation (the total serum mast cell tryptase should be > 1.2 × baseline tryptase + 2 μg/L) to diagnose acute mast cell activation (9). According to the above criteria, our patient showed significant acute mast cell activation with a serum tryptase level of 10.4 μg/L at onset of anaphylaxis and returned to a level of 2.7 μg/L 24 h later.

Most NSAID anaphylaxis is presumed to be immunoglobulin E (IgE)-mediated and is drug-specific, whereas non-immunologic anaphylaxis mainly involves direct mast cell and basophil activation and impaired metabolism of leukotrienes (10). Regardless of the pathogenesis, the clinical symptoms of both types of anaphylaxis are similar and often indistinguishable. Skin tests and/or specific IgE are helpful for identifying the cause of drug induced anaphylaxis. Screening for NSAIDs specific IgEs is not common available and skin prick testing should be carefully performed in those patients with histories of life-threatening anaphylaxis (3).

For NSAID-induced anaphylaxis, the BAT is suggested as a complementary diagnostic method that identifies the culprit agent with high probability. For NSAID-induced hypersensitivity, BAT is suggested as a good diagnostic method that is safer than drug provocation tests (11). BAT can identify the culprit drugs based on stimulation of whole blood cells with analgesics by measuring a basophil marker, CD63. A previous study reported that BAT for NSAID had sensitivity of 61%, specificity of 91%, positive predictive value of 92%, and negative predictive value of 59%. The reliability of BAT using another basophil activation marker, CD203c, was reported to have better sensitivity and specificity for NSAID-induced anaphylaxis (12). In conclusion, measurement of serial serum tryptase and BAT enabled us to diagnosis of anaphylaxis, determine the possible culprit drugs, and prevent further exposure to those drugs. Health professionals should educate the parents and children for allergy awareness and provide adrenaline autoinjectors for the emergency treatment of anaphylaxis.

## Data Availability Statement

The raw data supporting the conclusions of this article will be made available by the authors, without undue reservation.

## Ethics Statement

The studies involving human participants were reviewed and approved by Changhua Christian Hospital Institutional Review Board (IRB). Written informed consent to participate in this study was provided by the participants' legal guardian/next of kin.

## Author Contributions

Y-GT, W-YH, T-MC, and S-FK: had full access to all of the data in the study and takes responsibility for the integrity of the data and the accuracy of the data analysis. Y-GT and W-YH: drafting of the manuscript. Y-GT and W-HC: critical revision of the manuscript for important intellectual content. All authors contributed to the article and approved the submitted version.

## Funding

This work was supported in part by grants from the Ministry of Science and Technology, Taiwan, ROC (MOST107-2314-B-371-011-MY2), and from Changhua Christian Hospital (110-CCH-IRP-030, 110-CCH-ICO-152, 109-CCH-IRP-010, 108-CCH-IRP-051), and Academia Sinica, Taiwan (AS-SS-111-02-1).

## Conflict of Interest

The authors declare that the research was conducted in the absence of any commercial or financial relationships that could be construed as a potential conflict of interest.

## Publisher's Note

All claims expressed in this article are solely those of the authors and do not necessarily represent those of their affiliated organizations, or those of the publisher, the editors and the reviewers. Any product that may be evaluated in this article, or claim that may be made by its manufacturer, is not guaranteed or endorsed by the publisher.

## Abbreviations

BAT: Basophil activation test
ER: Emergency room
NSAID: Non-steroidal anti-inflammatory drugs.
